# A Case of Lepromatous Leprosy With Erythema Nodosum Leprosum

**DOI:** 10.7759/cureus.33846

**Published:** 2023-01-16

**Authors:** Lily Park, Carly E Wallace, Gabriella Vasile, Christopher Buckley

**Affiliations:** 1 Dermatology, Larkin Community Hospital, Miami, USA; 2 Osteopathic Medicine, Lake Erie College of Osteopathic Medicine, Bradenton, USA; 3 Dermatology, Goodman Dermatology, Roswell, USA

**Keywords:** multidrug antibiotic therapy, leprosy, mycobacterium leprae, erythema nodosum leprosum, immunologic reaction, lepromatous leprosy

## Abstract

Erythema nodosum leprosum is an immunologic reaction that occurs in patients with lepromatous leprosy. We present the case of a 23-year-old female with a one-week history of fever and painful erythematous nodules along her upper and lower extremities. The patient had immigrated to the United States from Micronesia, where she was partially treated for leprosy two years prior. Histological examination from a punch biopsy demonstrated noncaseating granulomatous inflammation with numerous bacilli highlighted by the Fite stain. The acid-fast bacilli smear was positive. Given the patient’s clinical, laboratory, and histological findings, a diagnosis of lepromatous leprosy with a type 2 erythema nodosum leprosum reaction was established. Multidrug antibiotic therapy with rifampin, dapsone, minocycline, and prednisone was initiated, following the addition of clofazimine. Early recognition and treatment of leprosy are crucial to preventing chronic and disabling complications, especially in instances of systemic inflammatory responses such as erythema nodosum leprosum.

## Introduction

Leprosy, also referred to as Hansen’s disease, is a chronic granulomatous disease caused by* Mycobacterium leprae*, which affects the skin and peripheral nerves. The prevalence of leprosy has decreased dramatically from 5.4 million cases in 1985 to less than 220,000 cases worldwide [1]. In the United States, 75% of new cases of leprosy occur in immigrants [2]. The transmission of *M. leprae* is not completely understood, but it is widely thought to occur through the respiratory route from the aerosols and droplets of infected individuals, as bacilli have been isolated from the nasal droplets of affected patients. Less often, leprosy may enter through eroded skin. There is also a growing amount of research suggesting that *M. leprae* can also be a zoonosis, as transmission has occurred through armadillos in the southern United States. The vast majority of exposed individuals do not develop the disease [3]. The incubation period between exposure and the cutaneous manifestation of the disease ranges from months to 30 years, with a mean length of four to 10 years for lepromatous leprosy [4].

Contrary to popular belief, leprosy is not highly contagious. Rather, there is approximately a 25% chance of acquiring the disease through household contact from individuals with lepromatous leprosy [5]. The additional risk factors associated with an increased risk of developing leprosy include advanced age, immunosuppression, genetic variations in the innate immune response, and armadillo exposure [6,7]. It is especially important to consider leprosy when encountering patients who have emigrated from endemic regions, such as South America, Africa, Southeast Asia, India, the Middle East, and Central America.

Although treatment is quite effective and leprosy is considered curable, early diagnosis and treatment are imperative to minimize the likelihood of developing sequelae, which are often irreversible and debilitating. First-line treatment consists of multidrug antibiotic therapy with rifampicin and dapsone, with or without clofazimine. Alternative pharmacologic options include minocycline, clarithromycin, or ofloxacin [8].

## Case presentation

**Figure 1:**
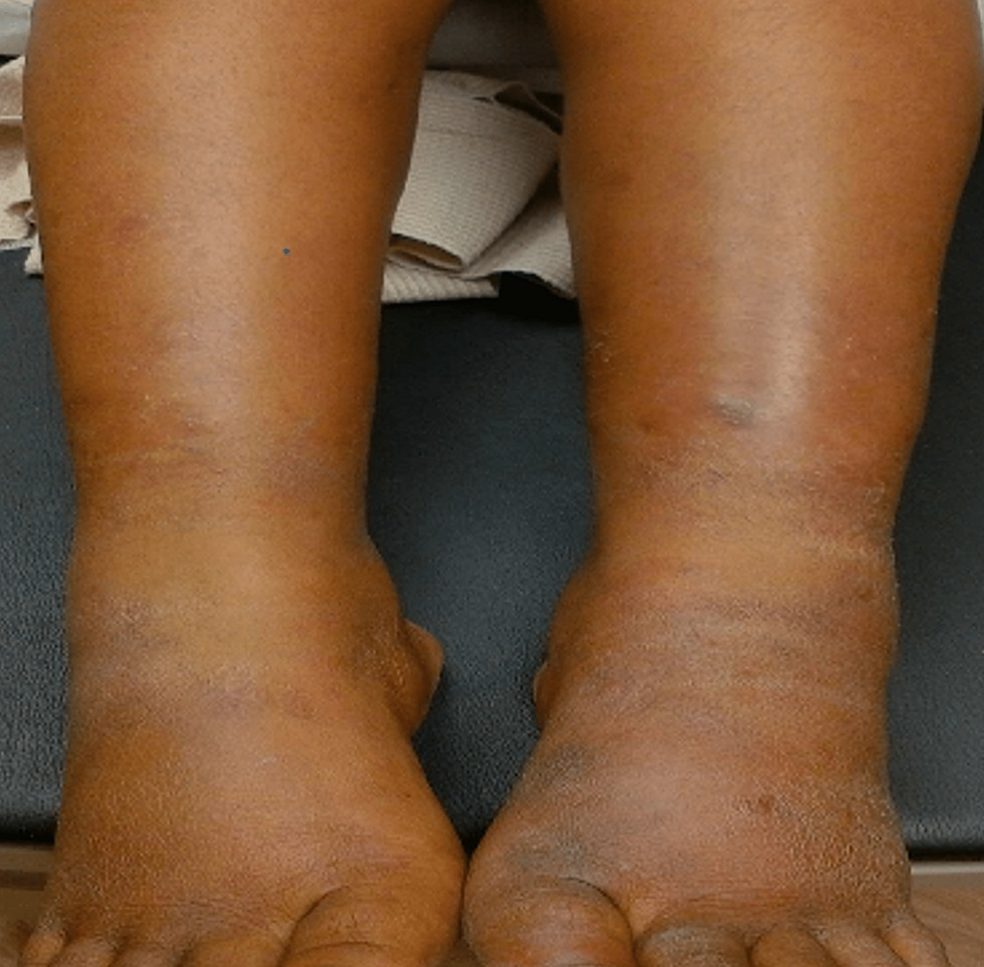
Edematous bilateral legs with hyperpigmented subcutaneous nodules varying in size from 0.5 to 2.0 cm

A 23-year-old female who had recently immigrated from Micronesia presented with a one-week history of painful nodules on her lower legs. She noted areas of decreased sensation throughout her upper and lower extremities. Associated symptoms included intermittent fever and night sweats. Previous treatment consisted of cephalexin for possible cellulitis, which was prescribed by her infectious disease physician, as well as empiric treatment with an unknown medication for nine months beginning two years prior from a leprosy clinic in Micronesia. Since relocating to the United States, she had discontinued all medications. Past medical history was negative for any immunodeficiencies. Physical examination revealed edema and erythema of the bilateral lower limbs with painful subcutaneous nodules of varying sizes. Hypopigmented macules were present on the upper arms, legs, and trunk (Figure 1).

**Figure 2:**
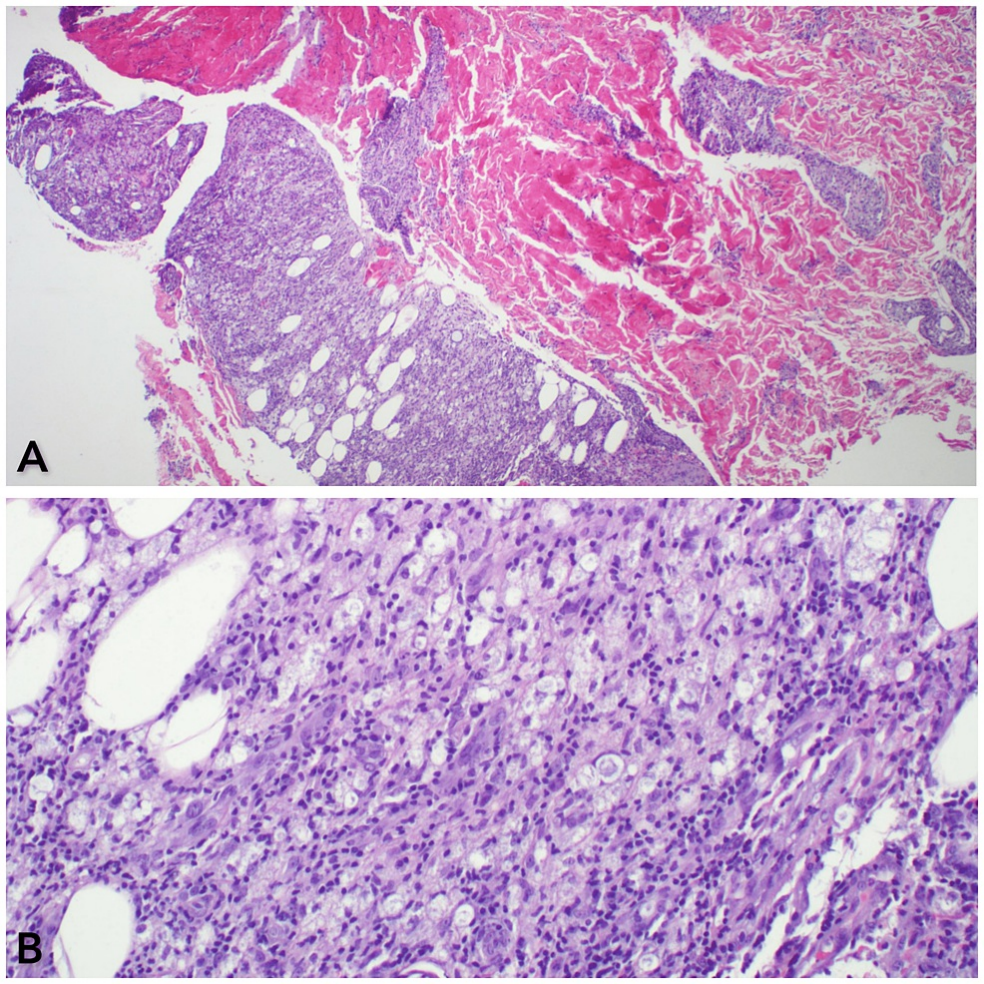
Hematoxylin and eosin staining showing noncaseating granulomatous inflammation with numerous bacilli, consistent with leprosy (A: magnification 4×, B: magnification 20×)

Laboratory evaluation was significant for leukocytosis and anemia. Quantiferon-TB Gold was negative. An 8 mm punch biopsy was performed along the lateral aspect of her left leg, which revealed noncaseating granulomatous inflammation with numerous bacilli highlighted by the Fite stain (Figure 2). The acid-fast bacilli smear was positive. Given the patient’s clinical, laboratory, and histological findings, a diagnosis of lepromatous leprosy with a type 2 erythema nodosum leprosum reaction was established.

Therapeutic management was initiated with rifampin, dapsone, folic acid, minocycline, and a short course of prednisone, which provided symptomatic relief. A trial of clofazimine was then administered following approval from her ophthalmologist.

## Discussion

Leprosy is an ancient disease dating back to ancient Egypt that predominantly occurs in resource-limited settings of endemic countries [9]. In the United States, leprosy remains a rare disease, with an incidence of 159 cases in 2020 [2]. The spectrum of clinical and histopathologic manifestations of leprosy is broad and can be divided into two major forms: tuberculoid and lepromatous leprosy. Ridley and Jopling established a classification system that reflects the entire continuum of histological, clinical, and immunologic features. The six categories consist of tuberculoid, borderline tuberculoid, mid-borderline, borderline lepromatous, lepromatous, and indeterminate [10].

Lepromatous leprosy presents acutely with generalized small, ill-defined macules early in the disease course, as was seen in our patient. When left untreated, these develop into numerous poorly demarcated, symmetric plaques or nodules along cooler areas of the body that may infiltrate the hair follicles. This most often occurs on the face, resulting in loss of eyebrows with “leonine facies” [11]. In severe cases, loss of body hair, nodular enlargement of the earlobes, and perforation of the nasal septum may occur [12]. Extracutaneous manifestations include peripheral neuropathy, predominantly manifesting as hypoesthesia or anesthesia of lesions, and ophthalmic injury [13].

Immunologic reactions are systemic inflammatory complications of leprosy that affect 30%-50% of all patients [14]. The two types of leprosy reactions are type 1 (also known as reversal reaction) and type 2 (also called erythema nodosum leprosum). Type 1 reaction occurs in patients with tuberculoid or borderline leprosy, while type 2 reaction presents as a sudden onset of painful erythematous nodules along the extensor surfaces of the extremities and face in patients with lepromatous leprosy. The pathogenesis of this reaction is incompletely understood but is attributed to the formation of immune complexes [3]. This reaction may develop throughout the natural course of the disease, during antimicrobial therapy, or following treatment completion [15]. The onset of erythema nodosum leprosum most often occurs within the first year of multidrug treatment [16]. Systemic manifestations include fever, arthralgias, tender lymphadenopathy, iridocyclitis, myalgias, and orchitis, among others [17]. Histopathology of erythema nodosum leprosum demonstrates histiocytes with many acid-fast bacilli and a chronic neutrophilic infiltrate [18].

The World Health Organization’s guidelines for the treatment of lepromatous leprosy involve multidrug management with dapsone, rifampicin, and clofazimine for 24 months [8]. Treatment should be continued should erythema nodosum leprosum develop. A short burst of prednisone is recommended for type 2 reactions to prevent irreversible nerve damage, with a gradual tapering once symptoms are controlled [19]. Thalidomide has considerable efficacy in men and women of nonreproductive potential; however, its use is limited due to teratogenesis [20]. Alternative agents for the management of erythema nodosum leprosum include pentoxifylline, methotrexate, azathioprine, infliximab, and etanercept [19]. For chronic control, clofazimine has demonstrated a protective role and is available through an investigational drug request to the Food and Drug Administration (FDA).

## Conclusions

The diagnosis of leprosy can be exceedingly difficult to recognize given its scarcity, variety of clinical presentations, and lengthy incubation period. The diagnosis of acute erythema nodosum leprosum is of particular importance, as there are numerous severe and debilitating consequences when left untreated, including claw hand, foot drop, lagophthalmos, and corneal ulceration. Therefore, physician awareness and prompt identification of lepromatous leprosy and its immunologic reactions are vital. Leprosy should be included as a differential diagnosis in all patients presenting with skin and neurological findings with any remote history of travel or immigration from endemic areas.
